# Incidence and risk factors of nocturnal penetrations and aspirations in patients with obstructive sleep apnea during drug‐induced sedation endoscopy

**DOI:** 10.1111/jsr.14314

**Published:** 2024-08-07

**Authors:** Igor Vainer, Raviv Allon, Yael Shapira‐Galitz, Lior Strinkovsky, Song Tar Toh, Shaun Loh, Uri Alkan

**Affiliations:** ^1^ Department of Otolaryngology Head and Neck Surgery Rabin Medical Center Petach‐Tikva Israel; ^2^ Sackler Faculty of Medicine Tel Aviv University Tel Aviv Israel; ^3^ Department of Otolaryngology, Head and Neck Surgery Kaplan Medical Center Rehovot Israel; ^4^ Faculty of Medicine Hebrew University of Jerusalem Jerusalem Israel; ^5^ Department of Otorhinolaryngology‐Head and Neck Surgery Singapore General Hospital Singapore Singapore; ^6^ Singapore SingHealth Duke‐NUS Sleep Centre Singapore Singapore

**Keywords:** aspirations, drug‐induced sleep endoscopy, obstructive sleep apnea, penetrations

## Abstract

Obstructive sleep apnea has been linked to an increased risk of pneumonia, possibly due to higher rates of nighttime aspirations. Few studies have directly investigated such aspirations in individuals with sleep apnea. This retrospective study included 142 adult patients with obstructive sleep apnea who underwent drug‐induced sedation endoscopy between 2017 and 2020. The incidence of penetrations and aspirations during the procedure was assessed, along with potential associated factors. The results showed that 28.1% of the patients experienced penetrations, 48.5% had aspirations, and 23.2% had neither. Male gender and epiglottic collapse were significantly associated with both penetrations and aspirations, while oropharyngeal collapse was more common in those without these events. This study highlights a high rate of aspirations during the procedure in individuals with sleep apnea, with epiglottic collapse and male gender identified as potential risk factors. These findings underscore the need for further research to understand the mechanisms of nighttime aspirations in sleep apnea and to develop targeted strategies to reduce pneumonia risk in this population.

## 1 INTRODUCTION

Obstructive sleep apnea (OSA) is a prevalent condition characterized by repetitive episodes of partial or complete upper airway obstruction during sleep, leading to disrupted breathing patterns and oxygen desaturation (Veasey & Rosen, 2019).

Beyond its immediate impact on sleep and oxygenation, OSA has been associated with a variety of comorbidities, including cardiovascular disease, diabetes and even chronic obstructive pulmonary disease (Huang et al., 2008). One particularly concerning association is the increased risk of pneumonia. Furthermore, a dose–response relationship has been observed, where increasing OSA severity is associated with a higher risk of developing pneumonia, as well as greater pneumonia severity and the need for more intensive medical interventions such as hospital admission, intensive care unit admission and invasive ventilation (Chiner et al., 2016; Lindenauer et al., 2014; Lutsey et al., 2023). Several hypotheses have been raised to explain the association between OSA and pneumonia, a leading one being reduced nocturnal swallowing safety. While a limited amount of microaspirations during sleep is normal (Ramsey et al., 2005), a higher rate of aspirations in patients with OSA is believed to be the leading mechanism for their increased risk of pneumonia (Beal et al., 2004; Su et al., 2014). Several explanations for this association have been suggested, including increased negative intrathoracic pressure built during apnea events causing a stronger pressure gradient and vacuum effect along the upper airway (Dempsey et al., 2010), decreased laryngeal sensation (Wallace et al., 2022), higher rate of gastroesophageal reflux disease (Kim et al., 2018; Morse et al., 2004), lower spontaneous deglutition rate (Sato et al., 2018), and impaired swallowing function and respiration coordination (Schar et al., 2021; Wang et al., 2016). However, compared with the vast amount of theoretical explanations, relatively few studies have demonstrated aspirations in this population.

The methodology of studying nocturnal swallowing patterns has been diverse and often indirect. Initially, studies examined nocturnal aspirations on a few patients using radiotracers that were poured during sleep via catheters installed in the nasopharynx and showed that all patients had some of the material aspirated, with a mean of 10% of the material aspirated in patients with OSA compared with 0.1% in normal patients (Beal et al., 2004). However, it is not possible to determine if the aspirations actually occurred during sleep, and it is not possible to rule out that the non‐physiological delivery mechanism of the radiotracer itself played a role in stimulating aspiration.

A few studies have utilized the Fibreoptic Endoscopic Evaluation of Swallowing (FEES) examination in patients with OSA to detect findings indicating oropharyngeal dysphagia, including spillage, retention and penetration in up to 64% of the cohort (Schindler et al., 2014). Other studies have shown higher Penetration‐Aspiration Scores (PAS) scores in patients with OSA (Pizzorni et al., 2021), and improvement in findings following the use of continuous positive airway pressure (CPAP; Caparroz et al., 2019). However, FEES is conducted in awake patients, which may underestimate the incidence of nocturnal aspiration.

To address this gap, two studies in the recent decade evaluated swallowing function during sedation. Patients without OSA undergoing elective gastrointestinal endoscopy were given coloured test liquid during endoscopic evaluation. Across both studies, aspirations were observed in up to 57.5% of participants, while penetrations were observed in up to 27.2% (Gemma et al., 2016; Liao et al., 2023).

This study aimed to investigate the incidence of penetrations and aspirations during sedation in patients with OSA undergoing drug‐induced sedation endoscopy (DISE). We hypothesized that patients with OSA will present a high incidence of penetration‐aspiration events. DISE is a diagnostic procedure utilized to assess upper airway collapse location and patterns in patients with OSA being considered for surgical intervention. While it is not intended for evaluation of swallowing, DISE allows a three‐dimensional and dynamic visualization of the upper airway during sleep‐like conditions (Dijemeni et al., 2017). Another objective to this study was to identify potential factors associated with nocturnal aspirations. We hypothesized that certain anatomical and physiological factors, such as the site of collapse and patient demographics, may predispose patients with OSA to these events.

## 2 METHODS

The study protocol was approved by the Institution Review Board Committee (0755‐19‐RMC).

### 2.1 Study population

In this retrospective study, records were extracted from the institute's electronic medical records (EMR) of all patients who underwent DISE procedure at a tertiary university‐affiliated medical centre between 2017 and 2020.

Included in this study were adult patients aged 18 years or older who were diagnosed with mild to severe OSA, and who failed CPAP treatment attempts.

Patients were excluded if they had known neurological disorders, including prior cerebrovascular accident, Parkinson's disease or neuromuscular disorders. Additionally, patients with a history of any previous intervention or radiation to the head and neck region, vocal fold disorders such as paralysis or immobility, complaints of dysphagia or insufficient data were excluded.

### 2.2 Outcome measures

The primary outcome measures of this study were the incidence of penetrations and aspirations of saliva during DISE. Penetration was defined as the presence of saliva in the laryngeal vestibule (the area bordered anteriorly by the epiglottis, posteriorly by the arytenoid cartilages, and laterally by the aryepiglottic folds) at or above the level of the true vocal folds.

Aspiration was defined as the presence of saliva distal to the true vocal cords during DISE. Penetrations or aspirations were considered according to the definitions above regardless of a preceding swallowing attempt.

A single observation of penetration or aspiration during an examination was sufficient for determining this outcome. If both penetration and aspiration were observed, the patient was included in the aspiration group.

Subjects were grouped into three groups according to the primary outcome measures: aspiration group; penetration group; and a non‐event group, which did not exhibit either penetrations or aspiration during the DISE.

Secondary outcomes included factors associated with penetrations and aspirations during DISE.

### 2.3 Data collection

All medical records were reviewed using our institute's EMR system (Chameleon, Elad Solutions, Tel‐Aviv, Israel).

Data collected included patient‐related general information, that is, demographic factors (age and sex) and smoking status (defined as at least five pack‐years described by the patient, or a smoker status documented in the EMR), physical examination findings including body mass index (BMI), neck and abdomen circumference, existence of nasal septum deviation, and oral cavity and pharyngeal examinations, including descriptions based on Brodsky, Mallampati, Friedman and Muller scales (Friedman & Hwang, 2015). Sleep examination findings were assessed by various sleep studies, including polysomnography, home partial sleep studies and overnight peripheral arterial tonometry (PAT) studies. Data collected from these studies included apnea–hypopnea index (AHI), respiratory disturbance index (RDI), and minimal O_2_ saturation. The Epworth Sleepiness Scale questionnaire is routinely filled in by our patients and its results were also collected.

Additional data included the site of collapse according to the DISE report: Vellum, Oropharynx, Base of tongue, and epiglottis. Collapse at a specific site was defined as binary (“yes” or “no”) based on a predefined 75% threshold of obstruction. Finally, peak propofol concentration in the blood was also collected from the DISE report.

### 2.4 Rating process

Rating of penetration‐aspirations as well as DISE obstruction sites was carried out by two independent sleep surgeons (authors U.A. and S.L.), with 8 and 10 years of experience in performing and interpreting DISE, respectively. Inter‐rater reliability was calculated for all examinations. Discrepancies were resolved by agreement. All raters were blinded to the patient's name and history.

### 2.5 DISE protocol

In our centre, patients are instructed to fast for at least 6 hr before the procedure. DISE is performed in a dimly lit operating room maintained at 22°C. The procedure is conducted by a team composed of an anaesthesiologist and otolaryngologist. Vital signs, including peripheral pulse oximetry, three‐channel electrocardiogram and non‐invasive blood pressure, are monitored at 3‐min intervals. Bispectral analysis and intravenous propofol is administered via a target‐controlled infusion (TCI) pump with an initial target plasma level of 2.0 μg ml^−1^. The dose is increased by 0.2 μg ml^−1^ every 2 min until the patient starts to snore, or vibration and collapse of the upper airway is observed. Once these occur, the duration of the procedure is 15 min. A flexible nasopharyngeal fibrescope (Olympus ENF‐GP2 rhino‐laryngo fibrescope; Olympus America, Center Valley, PA, USA; or PENTAX FNL‐10RBS; Pentax Canada, Mississauga, Ontario, Canada) is inserted through the nose following slight lubrication with 2% lidocaine gel. Additional instruments used for documentation were a high‐density camera (Karl Storz SCB Image 1 HD Hub 222010), cold light source (Karl Storz SCB xenon 300 20,134,020) and an integrated reception system (AIDA, Karl Storz Endoscopes GmbH and Co. KG, Tuttlingen, Germany).

Obstruction sites, configuration/obstruction pattern and degree/severity are classified according to established VOTE systems (Kezirian et al., 2011) and documented. Penetrations and aspirations are routinely recorded and documented according to the criteria mentioned in the study outcomes section as well as event of provoked cough reflex and awakening episodes.

### 2.6 Statistical analysis

Data were analysed using the SPSS statistical software version 25.0 (SPSS, Cary, NC, USA). Data distribution was analysed using Shapiro–Wilk test. For the analysis of continuous data, ANOVA test was used for normally distributed variables, and Kruskal–Wallis for non‐parametric variables. Chi‐square or Fisher's test were utilized for analysis of categorical variables. Inter‐rater reliability was assessed using Cohen's kappa statistic.

Finally, a multivariate analysis was performed in an attempt to determine predictors of penetrations and aspirations in patients with OSA during DISE. For this purpose, independent variables that reached a significance level of 0.05 in univariate analysis were included.

In all analyses, a two‐sided *p*‐value <0.05 was considered statistically significant. All presented means are accompanied by their respective standard deviations. A significance level of *p* < 0.05 was considered statistically significant.

## 3 RESULTS

**FIGURE 1:**
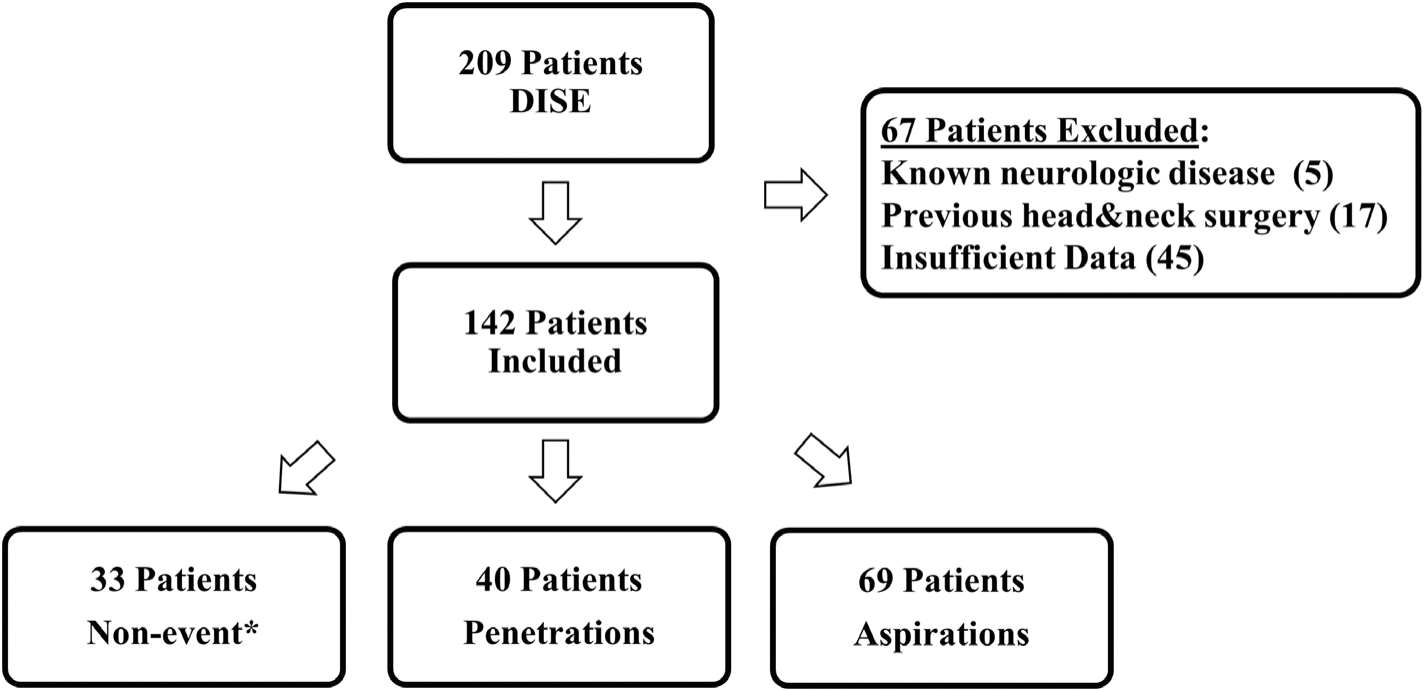
Cohort group distribution. A flowchart showing the study cohort distribution based on their DISE findings: aspirations, penetrations, or none. *Patients without observed penetrations or aspirations were defined as non‐event group. DISE, drug‐induced sleep endoscopy.

The study flowchart is presented in Figure 1. Two‐hundred and nine patients underwent DISE at our institution between 2017 and 2020. Of this cohort, 142 patients met the inclusion criteria and were included in the analysis. Forty‐five patients were excluded due to insufficient data, which included patients with missing information from the pre‐ or post‐DISE clinic visit and patients without videos of their DISE due to technical issues. The cohort comprised of 87.3% males, with a mean (standard deviation) age and AHI of 42.6 (± 11.6) and 26 (± 17.1), respectively.

Analysis revealed penetrations in 28.1% (*n* = 40) of the cohort, aspirations in 48.5% (*n* = 69), and 23.2% (*n* = 33) had neither aspirations nor penetrations (non‐event group). Seventeen patients, all from the aspiration group (24.6% of the aspiration group), had a cough reflex combined with short awakening. None of the penetration or non‐event groups coughed. The agreement rate between the two independent observers was 80%, indicating substantial agreement. Cohen's kappa test estimated the inter‐rater reliability at 0.7 (*p* < 0.001), which also suggests substantial agreement between the observers.

Patient characteristics are presented in Table 1. No significant differences were observed between groups in age, smoking status, physical examination, BMI or sleep test findings.

**TABLE 1:** Comparison of patient characteristics.

Parameters	Total, *n* = 142	Non‐event, *n* = 33	Penetrations, *n* = 40	Aspirations, *n* = 69	*p*‐Value
Age, years, mean ± SD	42.64 ± 11.65	41.76 ± 12.97	41.75 ± 9.52	43.58 ± 12.18	0.65
Sex (Male)	124 (87.3%)	25 (75.8%)	32 (80%)	67 (97.1%)	**0.003**
Smoking history	41 (28.9%)	11 (33.33%)	8 (20%)	22 (31.88%)	0.33
Physical examination					
BMI, mean ± SD	27.56 ± 4.12	26.85 ± 3.85	27.31 ± 3.78	28.02 ± 4.43	0.39
Abdominal circumference, mean ± SD	101.92 ± 13.12	104.79 ± 10.61	97.81 ± 13.09	103.26 ± 14.55	0.3
Neck circumference, mean ± SD	44.14 ± 4.78	43.15 ± 4.09	43.29 ± 5.96	45.04 ± 4.22	0.19
Deviated nasal septum	96 (67.6%)	21 (63.6%)	31 (77.5%)	44 (63.77%)	0.29
Brodsky scale > 2	27 (19.01%)	5 (15.15%)	5 (12.5%)	17 (24.64%)	0.24
Muller scale > 2	8 (5.63%)	3 (9.09%)	0 (0%)	5 (7.25%)	0.18
Friedman scale > 2	30 (21.13%)	5 (15.15%)	11 (27.5%)	14 (20.29%)	0.43
Mallampati scale > 2	40 (28.17%)	6 (18.18%)	16 (40%)	18 (26.09%)	0.1
Epworth Sleepiness Scale, mean ± SD	12.35 ± 5.65	13.44 ± 9.52	11.73 ± 3.95	12.16 ± 3.46	0.62
Propofol concentration, mean ± SD (μg ml^−1^)	2.58 ± 0.44	2.49 ± 0.34	2.69 ± 0.51	2.56 ± 0.051	0.42
Polysomnography					
AHI, mean ± SD	26.04 ± 17.1	24.29 ± 15.49	24.13 ± 17.39	28.13 ± 17.71	0.44
RDI, mean ± SD	30.08 ± 13.12	27.43 ± 14.38	27.75 ± 12.33	33.06 ± 12.57	0.36
Minimal O_2_ saturation, mean ± SD	84.02 ± 7.89	84.72 ± 5.26	84.79 ± 7.98	83.19 ± 8.99	0.54

*Note*: Bold values represent statistically significant variables.

Data are represented as *n* (%) unless otherwise indicated.

AHI, apnea–hypopnea index; BMI, body mass index; RDI, respiratory disturbance index; SD, standard deviation.

The only statistically significant difference between groups was observed for gender, with a male majority of 97.1% in the aspiration group compared with 80% and 75.8% for penetration and non‐event groups, respectively (*p* = 0.003).

The depth of anaesthesia was analysed by measuring the peak propofol concentration in the blood. Mean peak propofol concentration during sedation did not show differences between the mild, moderate and severe OSA severity group levels (2.62 [± 0.41], 2.53 [± 0.53] and 2.63 [± 0.45], respectively, *p* = 0.47), or between the non‐event, penetration and aspiration groups (2.49 [± 0.34], 2.69 [± 0.51], 2.56 [± 0.51], respectively, *p* = 0.42).

Table 2 presents the data regarding DISE outcomes. No significant difference was observed in velopharyngeal or base of tongue obstruction between groups. Oropharyngeal obstruction was less common in the aspiration group compared with the non‐event and penetration groups (60.8% versus 84.8% and 75%, respectively, *p* = 0.03). However, epiglottic collapse was more prevalent in the aspiration group, with over half (55%) of patients presenting with obstruction at this level compared with 36.3% in the non‐event group (*p* = 0.007).

**TABLE 2:** Intra DISE parameters.

Parameter	Total, *n* = 142	Non‐event (*n* = 33)	Penetrations (*n* = 40)	Aspirations (*n* = 69)	*p*‐Value
Site of collapse					
Vellum	137 (96.5%)	31 (93.94%)	38 (95%)	68 (98.55%)	0.42
Oropharynx	100 (70.4%)	28 (84.85%)	30 (75%)	42 (60.87%)	**0.03**
BOT	94 (66.2%)	19 (57.58%)	29 (72.5%)	46 (66.67%)	0.34
Epiglottis	60 (42.3%)	12 (36.36%)	10 (25%)	38 (55.07%)	**0.007**

*Note*: Bold values represent statistically significant variables.

Comparison of site of collapse during drug‐induced sleep endoscopy. Data are represented as *n* (%) unless otherwise indicated.

BOT, Base of tongue.

The results of the multivariate regression analysis for the predictors of penetrations and aspirations during DISE are presented in Table 3. In the multivariate analysis, male gender and epiglottic collapse remained significantly associated with penetrations and aspirations. However, oropharyngeal collapse lost significance in the multivariate analysis, and was only marginally associated with the absence of aspirations or penetrations.

**TABLE 3:** Multivariate analysis for factors predicting aspirations and penetrations.

Parameter	*χ* ^2^ ELRT^a^	Protective effect	*p*‐Value
Sex	16.02	Female	**0.0003**
Site of collapse			
Oropharynx	5.91		0.052
Epiglottis	11.05	No collapse	**0.004**

*Note*: Bold values represent statistically significant variables.

^a^ ELRT, effect likelihood ratio tests in a nominal logistic fit for aspiration group.

## 4 DISCUSSION

Our study aimed to investigate the incidence of aspirations and penetrations of saliva during DISE in patients with OSA, and identify their potential determinants. To the best of our knowledge, this is the first study to evaluate these phenomena. Our findings reveal that approximately half of patients with OSA experience aspirations during DISE, while an additional quarter experience penetrations without aspirations. These findings coincide with those demonstrated in two previous studies that evaluated swallowing functions under sedation in non‐OSA patients. In one study, Gemma et al. used TCI, and found a rise in penetration and aspiration rates that reached 57.5% and 27.2%, respectively, under target effect‐site propofol concentrations of 4 μg ml^−1^ (Gemma et al., 2016). In another study, Liao et al. used manually controlled infusion of propofol, and observed a 25.7% and 51.4% of penetrations and aspirations, respectively (Liao et al., 2023).

As mentioned above, it would have been expected that our cohort of patients suffering from OSA would present a higher incidence of penetrations and aspirations than healthy individuals with relatively similar mean age and gender distribution. One possible explanation is the difference in methodology between our study and previous studies. First, DISE is intended to observe obstruction patterns rather than dysphagia. Together with the retrospective design of our study, this might lead to underestimation of the penetration and aspiration events. Second, compared with previous studies that used coloured test liquid injected at the base of tongue (Gemma et al., 2016; Liao et al., 2023), our study observed saliva with no active injection of any material, which may also lead to underestimation compared with these studies.

Despite the hypothesis that patients suffering from OSA have a high rate of aspirations contributing to the increased risk of pneumonia (Beal et al., 2004; Su et al., 2014), there is also a possibility that there is no increased risk for penetrations and aspirations in patients with OSA during sedation, as our comparison to a previous study has shown. Another piece of evidence supporting this is the univariate analysis performed in this study that did not show any association between OSA severity and the incidence of aspirations.

An unanswered question is the association between the penetrations‐aspirations seen during DISE/sedation and those occurring during natural sleep. Sedation does not accurately imitate natural sleep. it minimizes and frequently abolishes rapid eye movement (REM) stage of sleep in favour of the N3 stage (Rabelo et al., 2013). Whether this alters the rate of aspirations is still unanswered. However, it is likely that the difference may be major. For instance, Sato et al. (2018). explored deglutition patterns during natural sleep in patients with OSA. They observed 0.6 deglutition during N3 sleep compared with none in REM sleep. Nocturnal deglutition are commonly followed by inspiration and thus are considered a precursor of aspiration.

Kohno et al. (2022) used hypothesized surrogate markers of aspiration during polysomnography such as presence of swallows detected by electromyography followed by coughs detected by a night‐vision camera, to identify potential aspirations. They found a mean of 1.7 (SD 2.1) and a median of 1 (confidence interval [CI] 0–2.3) aspiration events per night in patients with OSA, with no significant differences from non‐OSA patients. It is difficult to compare these results with the ones in our study or previously discussed studies as we observed the incidence of aspirations rather than the amount per night. Nevertheless, Kohno et al.'s methodology likely underestimates the true aspiration rate because their measurement relies on indirect markers such as cough and swallowing, potentially missing silent aspirations reported in the range of 10%–100% by other studies (Gleeson et al., 1997; Huxley et al., 1978; Kikuchi et al., 1994; Ozcan et al., 2003).

In addition, propofol is known to cause hypersalivation (Kang et al., 2008) and to lower pharyngeal muscles tone (Rimaniol et al., 1994; Sundman et al., 2001), which may lead to an overestimation of aspirations in DISE/sedation conditions compared with natural sleep. Furthermore, the local anaesthetic effects of the lidocaine gel need to be considered, though with the minute amount used it is very unlikely that the lidocaine gel applied to the fibreoptic laryngoscope affected the examination (Kamarunas et al., 2014).

We found a few risk factors for aspiration during DISE. Epiglottic collapse was observed in more than half of the aspiration group, significantly more than in the penetration or non‐event groups. Previous studies (Lan et al., 2015) reported similarly high rates of epiglottic collapse, but they did not examine its association with aspiration. When the pharyngeal phase of the swallowing reflex starts, the larynx elevates, and the epiglottis covers the laryngeal vestibule, a mechanism that is well coordinated with respiration (Matsuo & Palmer, 2008). A possible explanation for the possible association with epiglottic collapses and aspirations is that a weak and floppy epiglottis may cause airway obstruction, leading to pooling of saliva around it. When the obstruction resolves, the accumulated saliva near the epiglottis, in close proximity to the airway, combined with the high negative intrathoracic pressure following apnea, all contribute to an increased risk of aspiration.

Given its proximity to the airway, we expected a similar association with base of tongue obstruction. While higher rates of this obstruction were observed in the aspiration group, the difference was not statistically significant.

The proximity to the airway may also explain the protective effect of oropharyngeal obstruction against aspirations observed in this study. Our results show that oropharyngeal obstruction was more common in the non‐event group than in the penetration and aspiration group. Compared with the epiglottis, pooling of saliva in the oropharyngeal level anterior to the palatine tonsils may leave enough time and space for the swallowing reflex to clear the accumulated saliva properly and avoid penetration to the larynx. However, our results imply that the risk caused by epiglottic obstruction may play a more critical role than the protective effect of oropharyngeal obstruction, as implied by the multivariate analysis.

Another risk factor for saliva aspirations during DISE demonstrated in this study was male gender. Despite the known higher rate and severity of OSA in males (Wahner‐Roedler et al., 2007), previous studies have also shown differences in the pathophysiology of OSA between genders. For example, despite having greater luminal area (Mohsenin, 2001; Ye et al., 2009), men's pharyngeal airways are more collapsible than those of females when exposed to greater intraluminal pressure (Mohsenin, 2003; Pillar et al., 2000). Studies indicate that men generate almost twice as much pressure as women to achieve similar peak flow rates (Trinder et al., 1997). The high negative pressure required to maintain pharyngeal airway patency may adversely lead to the suctioning of saliva into the larynx, potentially explaining the higher rates of aspirations seen in men in our study. Surprisingly, our study did not show association between OSA severity and the rate of aspirations during DISE; a connection between the variables would have been expected, especially when previous studies showed an association between pneumonia rate and severity to OSA severity (Chiner et al., 2016; Lutsey et al., 2023). This could be explained by the fact that our cohort was limited mostly to patients with moderate to severe OSA and intolerant to CPAP, which could introduce bias toward more severe cases. However, this lack of association does suggest that the higher aspiration rates in men stem from the differences in the pathophysiology of the disease between genders rather than differences in the severity of the disease.

Adding to the inherent limitations of a retrospective study, other limitations of our study include the subjective assessment of collapse location and pattern, as well as aspirations and penetrations during DISE that may vary between observers, despite efforts to minimize bias through independent analysis and adjudication. Discussed earlier are the limitations involved in examining swallowing functions via DISE, which is intended for the observation of collapse patterns. It is also important to note that there is a variability in the sedation level between patients. Our DISE protocol states that procedure begins when the patient starts to snore, or vibration and collapse of the UA is observed, which may be seen at different doses between patients. Moreover, while the study controlled for several variables, there may be unmeasured or residual confounding factors that could influence the outcomes, such as dietary habits, alcohol consumption or other medical conditions.

Further prospective studies focusing on evaluating penetration‐aspiration during sedation, following the methodology of Gemma et al.'s study (Gemma et al., 2016), but in patients with OSA, could help address these issues.

## 5 CONCLUSION

Our study reveals that a significant proportion of patients with OSA experience aspirations of saliva during DISE, with a notable number also experiencing penetrations without aspirations. Our findings suggest that epiglottic collapse may be linked to a higher risk of aspirations, while oropharyngeal collapse could have a protective effect. Additionally, our study indicates that men may be at a higher risk of aspirations, possibly due to physiological differences between genders rather than the severity of the disease.

## AUTHOR CONTRIBUTIONS

**Igor Vainer:** Writing – original draft; writing – review and editing; methodology; data curation. **Raviv Allon:** Writing – original draft; writing – review and editing. **Yael Shapira‐Galitz:** Validation; writing – review and editing. **Lior Strinkovsky:** Investigation; resources. **Song Tar Toh:** Validation; formal analysis. **Shaun Loh:** Validation; formal analysis. **Uri Alkan:** Conceptualization; methodology; supervision.

## FUNDING INFORMATION

None.

## CONFLICT OF INTEREST STATEMENT

The authors have no conflicts of interest to disclose.

## Data Availability

The data that support the findings of this study are available on request from the corresponding author. The data are not publicly available due to privacy or ethical restrictions.
